# Transcriptome-Wide Analysis of RNA N^6^-Methyladenosine Modification in Adriamycin-Resistant Acute Myeloid Leukemia Cells

**DOI:** 10.3389/fgene.2022.833694

**Published:** 2022-04-28

**Authors:** Shu Fang, Bo Peng, Yanan Wen, Jingjing Yang, Hao Wang, Ziwei Wang, Kun Qian, Yan Wei, Yifan Jiao, Chunji Gao, Liping Dou

**Affiliations:** ^1^ School of Medicine, Nankai University, Tianjin, China; ^2^ Department of Hematology, the Fifth Medical Center of Chinese PLA General Hospital, Beijing, China; ^3^ Medical School of Chinese PLA, Beijing, China

**Keywords:** acute myeloid leukemia, drug resistance, N^6^-methyladenosine, gene expression, METTL3

## Abstract

Acute myeloid leukemia (AML) is one of the most aggressive hematopoietic malignancies. Patients still suffer from refractory/relapsed disease after anthracycline-based therapy, which leads to a poor prognosis. N^6^-Methyladenosine (m^6^A) is the most abundant post-transcriptional modification in eukaryotes, the imbalance of which is reported to be associated with various pathological processes, including drug resistance. However, the relationship between m^6^A modification and drug resistance has not been well defined in AML. In this study, we analyzed the sequencing data of HL60 and its Adriamycin-resistant cell line HL60/ADR. We found a total of 40,550 m^6^A-methylated peaks, representing 15,640 genes in HL60, and 38,834 m^6^A-methylated peaks, representing 15,285 genes in HL60/ADR. KEGG pathway analysis showed that pathways were enriched in the FoxO signaling pathway, p53 signaling pathway, and Notch signaling pathway. MeRIP-seq results showed that the fold enrichment of the global m^6^A level in HL60/ADR was higher than that in HL60, and dot blot assay results indicated that the global m^6^A level was elevated in HL60/ADR cells compared with that in HL60 cells. Further analysis revealed that the expression level of METTL3 was elevated in HL60/ADR cells compared with that in HL60 cells. After a combined treatment of STM2457 (an inhibitor of METTL3) and Adriamycin, the proliferation of HL60/ADR was inhibited. Thus, we hypothesized that the abnormality of m^6^A modification played an important role in Adriamycin-resistant AML.

## Introduction

Acute myeloid leukemia (AML) is one of the most common and aggressive hematopoietic malignancies with complex pathogenesis (Reilly, 2005; Yamamoto and Goodman, 2008; Zhou et al., 2017; Dou et al., 2019a; Dou et al., 2019b). Anthracycline-based therapy remains the major induction therapy for AML patients. However, approximately 20–30% of patients still suffer from a refractory disease, and the majority of patients relapse after achieving complete remission (CR) (Dombret and Gardin, 2016; Döhner et al., 2017; Schlenk et al., 2017; Estey, 2018). The mechanism for anthracycline resistance in AML patients is reported to be associated with abnormal activation of pathways and epigenetic modification, which are rather complex (Wang et al., 2019; Lin et al., 2020; Wang et al., 2020). A further study is still necessary to figure out the mechanism for anthracycline resistance to improve the prognosis of patients with refractory/relapsed AML.

N^6^-Methyladenosine (m^6^A) is the most abundant post-transcriptional modification in eukaryotes, which existed at the consensus motif of RRm^6^ACH (where R denotes A/G and H denotes A/C/U) (Fu et al., 2014). M^6^A modification has been reported to play an important role in tumorigenesis (Jin et al., 2019; Yue et al., 2019; Chen and Wong, 2020; Shen et al., 2020). Some studies have reported that m^6^A modification, which is critical for post-transcriptional regulation of gene expression, is mainly enriched around stop codons, 3′ untranslated region (UTR), and codon regions of mRNA (Rana and Tuck, 1990; Meyer et al., 2012). M^6^A modification is dynamically regulated by m^6^A writers, which deposit m^6^A in RNA, and m^6^A erasers, which demethylate m^6^A from RNA, while m^6^A readers can identify the m^6^A modification and determine the ultimate fate of RNA—translation or degradation (Meyer and Jaffrey, 2017; Zhang et al., 2020). These m^6^A regulators play key roles in maintaining normal stem cell hematopoiesis and other physiological and pathological processes (Karthiya and Khandelia, 2020; Wang et al., 2021), and the imbalance of these m^6^A modification regulators may result in hematological malignancies (Zhao and Peng, 2022). M^6^A regulators containing METTL3, METTL14, WTAP, YTH domain-containing proteins, IGF2BP family members, FTO, and ALKBH5 have been reported to be up-regulated in AML. Depletion of these regulators in AML may induce leukemia differentiation and apoptosis (Yankova et al., 2021). These findings reveal the possibility of m^6^A regulators as potential therapeutic targets for AML.

To date, the role of m^6^A has not been fully investigated in anthracycline-resistant AML. To study the global m^6^A modification pattern in anthracycline-resistant AML, we analyzed the sequencing data of AML cell line HL60 and its Adriamycin (ADR)-resistant cell line HL60/ADR. To further study whether m^6^A regulators were dysregulated in HL60 and HL60/ADR cells, we used real-time quantitative polymerase chain reaction (RT-qPCR) and Western blot assay to analyze the expression levels of m^6^A writers and erasers. Then, we explored their prognosis in AML patients in TCGA database. In this study, we hypothesized that m^6^A modification plays an important role in ADR resistance.

## Methods

### Cell Line and Cell Culture

Human AML cell line HL60 and its ADR-resistant cell line, HL60/ADR, were gifted by the Tianjin Institute of Hematology. In brief, HL60 cells in a 100-mm dish were treated with a low dose of ADR to remove the drug-sensitive cells until their growth rate was similar to the untreated counterpart. Then, the concentration of ADR was increased stepwise to 0.5 μg/ml (Wang et al., 2019). HL60 cells were cultured in the RPMI-1640 medium supplemented with 10% fetal bovine serum (FBS), 100 μg/ml penicillin, and 100 μg/ml streptomycin at 37°C in a humidified 5% CO_2_ atmosphere. HL60/ADR cells were cultured under the same condition with additional ADR added to the medium at a final concentration of 0.5 μg/ml to maintain ADR resistance. The cells were cultured in an ADR-free culture medium for a week to eliminate ADR before the following experiments.

### mRNA Extraction and Dot Blot Assay

The cells were collected for total RNA extraction by adding 1 ml TRIzol reagent (Invitrogen, Carlsbad, CA, United States). Trichloromethane (UN No. 1888, Beijing, China), isopropyl alcohol (UN No. 1823, Beijing, China), and 75% ethanol (UN No.1170, Beijing, China) were used for RNA separation, precipitation, and purification. mRNA was extracted using GenElute™ mRNA Miniprep Kit (MRN70-1KT, Sigma, United States) following the manufacturer’s procedure. In brief, oligo (dT) beads were added to the 2 μg RNA samples and vortexed thoroughly and then incubated in a metal bath at 70°C. After centrifuging, RNA samples were washed twice in the wash solution and eluted twice in the elution solution. Finally, mRNA was obtained for dot blot assay. After denaturation, RNA was loaded onto an Amersham Hybond-N+ membrane (GE Healthcare, United States) preassembled on a bio-dot microfiltration apparatus (170-3938, Bio-Rad, United States) and washed with RNase-free water in advance. Then, the membrane was removed from the apparatus and washed once. RNA was cross-linked to the membrane by 80°C in the oven for 2 h. The membrane was blocked with 5% non-fat dry milk for 2 h, incubated with a specific anti-m^6^A antibody diluent (1:500 dilution, 61,495, Proteintech, United States) overnight at 4°C, and then incubated with HRP-conjugated anti-rabbit IgG (7074S, CST, United States) for 1 h at room temperature. At last, the membrane was developed with Clarity Western ECL Substrate (170-5060, Bio-Rad, United States).

### RT-qPCR

Total RNA was isolated by TRIzol Reagent. PrimeScript™ RT reagent kit (RT001, Takara, Japan) was used to synthesize cDNA according to the manufacturer’s procedure. Then, real-time qPCR was performed by using KAPA SYBR FAST q-PCR Master Mix (2x) Kit (KK4601, KAPA, United States). Specific primers for each evaluated gene are listed in Supplementary Table S1.

### Western Blot Assay

Over 10^6^ cells were harvested and washed twice. Proteins were extracted in a RIPA lysis buffer (#R0020, Solarbio, China) with the addition of PMSF (No. P0100, Solarbio, China) and protein phosphatase inhibitor (P1260, Applygen, China). Then, proteins were separated by SDS polyacrylamide gel electrophoresis (SDS-PAGE) and transferred onto a polyvinylidene difluoride membrane. After that, the membrane was blocked with 5% non-fat dry milk for 2 h at room temperature. The membrane was incubated with a specific anti-METTL3 antibody (86132S, CST) and anti-GAPDH antibody (sc-47724, Santa Cruz Biotechnology, United States) at 4°C overnight. After washing, the membrane was incubated with HRP-conjugated anti-rabbit IgG (7074S, CST, United States, anti-METTL3 antibody) and HRP-conjugated anti-mouse antibody (A0216, Beyotime, China, anti-GAPDH antibody) at room temperature for 40 min. At last, the membrane was developed with Clarity Western ECL Substrate (170-5060, Bio-Rad, United States).

### Cell Counting Kit-8 Assay

Cells were seeded in 6-well microplates at a density of 10^5^ cells/well and treated with 0.5 μg/ml ADR and different concentrations of STM2457 (HY-134836, MCE, United States, an inhibitor of METTL3, 0, 5, 10, 20 μmol/L). On day third, cells were harvested and washed with phosphate buffer saline (PBS) and seeded on a 96-well plate. Then, 100 ul culture medium and 10% CCK-8 solution were added to each well. Cells were incubated at 30°C for 2 h. The 96-well plate was then put into a microplate reader (Thermo, United States) to detect the optical density (OD) at 450 nm. The OD value of HL60/ADR was divided by that of HL60 to obtain the ratio of viable cells, as published previously (Han et al., 2015).

### MeRIP-Seq and RNA-Seq

Sample preparation was performed at LC-Bio Technology co., Ltd. (Hangzhou, China). Total RNA was extracted as described above. The RNA amount and purity of each sample were quantified using NanoDrop ND-1000 (NanoDrop, Wilmington, DE, United States). The RNA integrity was assessed by Bioanalyzer 2100 (Agilent, CA, United States) with RIN number >7.0 and confirmed by electrophoresis with denaturing agarose gel. Poly (A) RNA was purified from 50 μg total RNA using Dynabeads oligo (dT) 25-61005 (Thermo Fisher, CA, United States) using two rounds of purification. The poly(A) RNA was fragmented into small pieces using Magnesium RNA Fragmentation Module (NEB, cat. e6150, United States) at 86°C for 7 min and then incubated at 4°C for 2 h with an m6A-specific antibody (No. 202003, Synaptic Systems, Germany) in IP buffer (50 mM Tris–HCl, 750 mM NaCl and 0.5% Igepal CA-630). After that, the IP RNA was reverse-transcribed to create the cDNA by SuperScript™ II Reverse Transcriptase (1896649, Invitrogen, United States), which were next used to synthesize U-labeled second-stranded DNAs with *E. coli* DNA polymerase I (m0209, NEB, United States), RNase H (m0297, NEB, United States) and dUTP solution (R0133, Thermo Fisher, United States). An A-base is then added to the blunt ends of each strand, preparing them for ligation to the indexed adapters. Each adapter contains a T-base overhang for ligating the adapter to the A-tailed fragmented DNA. Single- or dual-index adapters are ligated to the fragments, and size selection was performed using AMPureXP beads. After the heat-labile UDG enzyme (NEB, cat.m0280, United States) treatment of the U-labeled second-stranded DNAs, the ligated products are amplified with PCR under the following conditions: initial denaturation at 95°C for 3 min; 8 cycles of denaturation at 98°C for 15 s, annealing at 60°C for 15 s, and extension at 72°C for 30 s; and then final extension at 72°C for 5 min. The average insert size for the final cDNA library was 300 ± 50 bp. At last, 2 × 150 bp paired-end sequencing (PE150) was performed on an Illumina Novaseq™ 6000 following the vendor’s recommended protocol. Two replicates in each IP and input sample were subjected to sequencing.

### MeRIP-qPCR

Total RNA was extracted and fragmented. A portion of RNA was labeled as the input control. The rest was used for immunoprecipitation (IP) and incubated with anti-m^6^A antibody-coupled beads. Then, RNA was immunoprecipitated and eluted. Both input and IP parts were subjected to RT-qPCR. Five genes with differentially methylated peaks were selected. Gene-specific qPCR primers are shown in Supplementary Table S2.

### Data Analysis

#### MeRIP-Seq and RNA-Seq Analysis

Fastp software (https://github.com/OpenGene/fastp) was used to control the quality of raw data in both IP and input samples. HISAT2 (http://daehwankimlab.github.io/hisat2) was used to map reads to the reference genome Homo sapiens (Version: v101, ftp://ftp.ensembl.org/pub/release-101/fasta/homo_sapiens/dna/). ExomePeak (http-s://bioconductor.org/packages/exomePeak) of R packages was used to perform peak calling analysis and differential peak analysis, and visualization was performed by IGV software (http://www.igv.org). Peak calling analysis was performed with the selection criteria of *p* < 0.05 between IP and input samples. Differentially methylated peaks were defined as fold change >2 and *p* < 0.05. ChIPseeker (https://bioconductor.org/packag-es/ChIPseeker) was used to annotate called peaks. MEME (http://meme-suite.org) and HOMER (http://homer.ucsd.edu/homer/motif) were used for *de novo* and known motif findings. StringTie (https://ccb.jhu.edu/software/stringtie) was used to calculate FPKM (total exon fragments/mapped reads (millions) × exon length (kB)) for all mRNAs from input libraries. The differentially expressed mRNAs were analyzed by edgeR of R packages (https://bioconductor.org/packages/edgeR) with the selection criteria of log2 (fold change) > 1 or log2 (fold change) ≤1 and *p* < 0.05. Data were analyzed using the R 3.5.2 software at LC-Bio Technology co. Two replicates in each group were analyzed.

### Sequence Statistics and Quality Control

After the raw data were obtained, reads with adaptor were removed, reads containing more than 5% of N were removed (N indicated that base information cannot be determined), and low-quality reads were removed (low-quality meant more than 20% of the total reads with a mass value of Q ≤ 10). Samples and quality control information are shown in Supplementary Table S3.

### Statistical Analysis

Values of two replicates in each sample were averaged for subsequent analysis. M^6^A peaks were defined as fold change >2 and *p*<0.05 between IP and input samples. Fold enrichment of m^6^A peaks (Figures 2F,G) and overall gene expression level (Figures 4A,B) were exhibited as quartile and range. Mann–Whitney U test was used for comparison between the two groups. Kruskal–Wallis test was used for comparison among three or more groups. The expression level of m^6^A-related enzymes by RNA-seq was presented as the mean (Figure 3G). RT-qPCR results were presented as mean ± SD (Figure 5B). The Student’s t-test was used to perform comparisons. GraphPad Prism 8.0 software was used for the abovementioned analysis.

### TCGA Dataset and Data Analysis

Clinical information and gene expression information of AML patients were downloaded from the TCGA database. Kaplan–Meier method and log-rank test were used to analyze the relationship between gene expression level and overall survival (OS) by R version 4.0.3. SPSS 24.0 was used to compare patients’ characteristics. Continuous variables were shown as median (range). Mann–Whitney U test was used for the comparison. The chi-squared test and Fisher’s exact test were used to compare categorical variables. A two-sided *p* < 0.05 was considered statistically significant.

## Results

### Global m^6^A Modification Patterns in HL60 and HL60/ADR Cells

**FIGURE 1:**
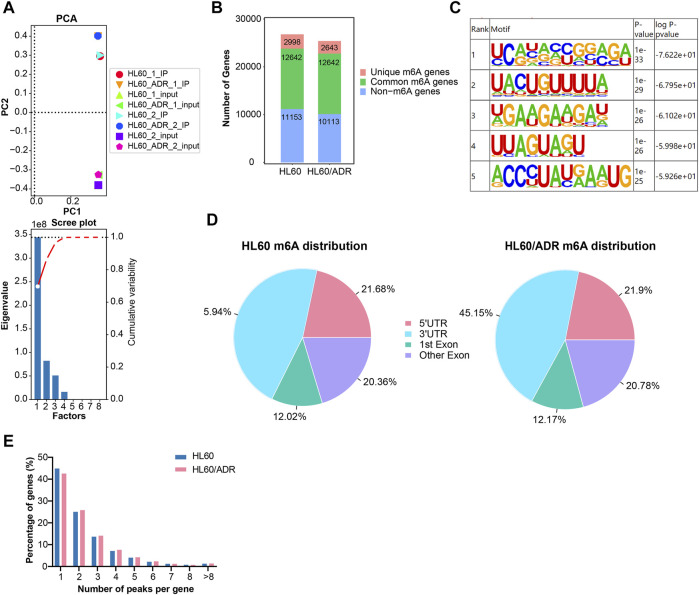
Overview of m^6^A modification patterns in HL60 and HL60/ADR cells. **(A)** PCA analysis revealed that biologically duplicated samples of HL60 and HL60/ADR were comparable. PCA, principal component analysis. **(B)** Distribution of m^6^A-modified genes and non-m^6^A-modified genes in HL60 and HL60/ADR. **(C)** Top five conserved motifs identified from m^6^A peaks shared RRACH consensus sequences in both HL60 and HL60/ADR. **(D)** Enrichment of m^6^A peaks in different regions of transcripts, and m^6^A peaks were mostly enriched in 3′ UTR and CDS. UTR: untranslated region, CDS: coding sequence. **(E)** Percentage of genes harboring a different number of m^6^A peaks.

HL60 and HL60/ADR cells were collected to perform methylated RNA immunoprecipitation combined with next-generation sequencing (MeRIP-seq). Principal component analysis (PCA) showed that IP and input samples of HL60 or HL60/ADR cells were comparable (Figure 1A).

M^6^A genes were defined as genes with m^6^A methylated peaks, and non-m^6^A genes were defined as genes without m^6^A methylated peaks. The number of m^6^A genes could be acquired by MeRIP-seq results of IP versus input samples, whereas non-m^6^A genes could only be detected in input samples. In our study, a total of 40,550 m^6^A methylated peaks representing 15,640 genes were detected in HL60, and 38,834 m^6^A methylated peaks representing 15,285 genes were detected in HL60/ADR. There were only 2998 genes with 23,004 m^6^A methylated peaks detected in HL60 and 2643 genes with 21,281 m^6^A methylated peaks found in HL60/ADR. There were 12,642 genes with 17,546 m^6^A methylated peaks detected in HL60 and 12,642 genes with 17,553 m^6^A methylated peaks detected in HL60/ADR (Figure 1B). Motif enrichment analysis showed that conserved motifs identified from m^6^A peaks shared RRACH consensus sequences in both HL60 and HL60/ADR (Figure 1C) (Fu et al., 2014).

To analyze transcriptome-wide m^6^A distribution in two cell lines, we divided m^6^A methylated peaks into 5′ untranslated region (UTR), coding sequence (CDS, including first exon and other exons), and 3′ UTR according to their locations in RNA transcripts. All in all, peaks were mainly enriched in CDS and 3′UTR of mRNA in both cell lines (Figure 1D). In our study, peaks with fold enrichment (FC) ≥2 and *p*<0.05 were defined as m^6^A peaks. Genes with one to four m^6^A peaks accounted for 90.57% (13,320/14,707) of all genes with m^6^A peaks in HL60 and 90.12% (13,161/14,604) of all genes with m^6^A peaks in HL60/ADR. About 44.8% (6596/14,707) and 42.5% (6213/14,604) of all m^6^A-modified genes had the unique m^6^A peak in HL60 and HL60/ADR, respectively (Figure 1E).

### Analysis of Differentially m^6^A-Modified Genes and m^6^A Peaks

**FIGURE 2:**
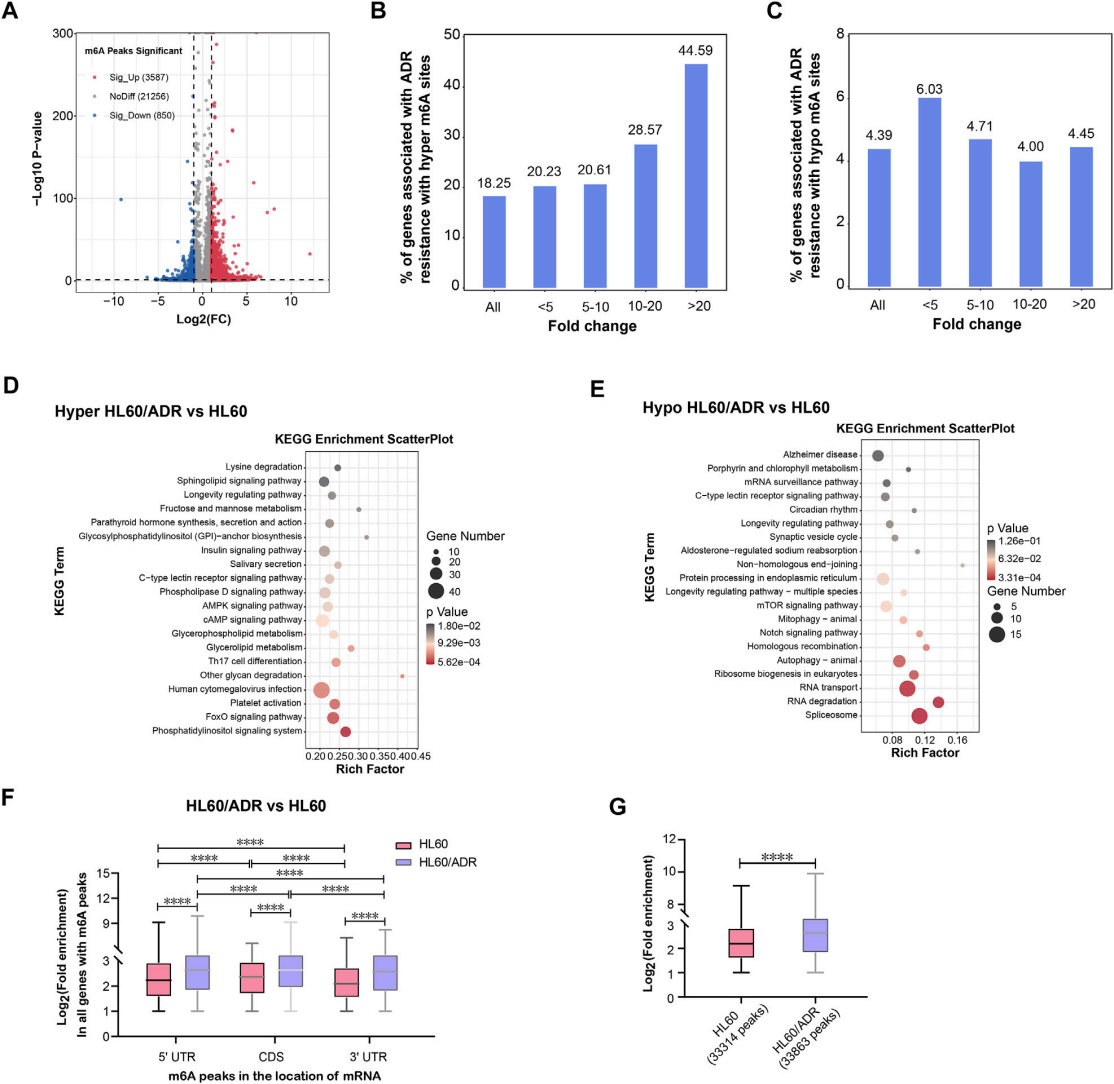
Analysis of differentially methylated m^6^A-modified mRNAs. **(A)** Volcano plot of differential m^6^A peaks. Red and blue dots represent hyper-methylated and hypo-methylated peaks, respectively. **(B)** Percentage of hyper-methylated genes in all genes with m^6^A modification in HL60/ADR cells. **(C)** Percentage of hypo-methylated genes in all genes with m^6^A modification in HL60/ADR cells. **(D,E)** KEGG pathway analysis of genes with hyper- **(D)** or hypo-methylated **(E)** m^6^A peaks in HL60/ADR compared with HL60. Rich factor, significant gene number/background gene number. KEGG, Kyoto Encyclopedia of Genes and Genomes. **(F)** Fold enrichment of all m^6^A peaks in different locations of mRNA. *p<0.05, ****p<0.0001. NS, no significance. **(G)** Comparison of fold enrichment of all m^6^A peaks in HL60 and HL60/ADR. ****p<0.0001.

A total of 4,437 differentially methylated m6A peaks within 3,461 genes have been found between HL60 and HL60/ADR. Among them, 3,587 differential m^6^A peaks within 2,790 genes were hyper-methylated, and 850 m^6^A peaks within 671 genes were hypo-methylated (Table 1; Figure 2A). In general, up-regulated methylated m^6^A-modified genes accounted for 18.25% of m^6^A-labeled genes in HL60/ADR, and down-regulated methylated m^6^A-modified genes accounted for 4.39% of m^6^A-labeled genes in HL60/ADR. Up-regulated methylated m^6^A-modified genes with fold enrichment of m^6^A-methylated peaks at <5, 5–10, 10–20, and >20 accounted for 20.23, 20.61, 28.57, and 44.59% of m^6^A-labeled genes, respectively, in HL60/ADR. Down-regulated methylated m^6^A-modified genes with fold enrichment of m^6^A-methylated peaks at <5, 5–10, 10–20, >20 accounted for 6.03, 4.71, 4.00, and 4.45% of m^6^A-labeled genes, respectively, in HL60/ADR (Figures 2B,C). The top ten up-regulated and down-regulated methylated m^6^A-modified genes with the highest fold enrichment are shown in Tables 2, 3, of which 4 out of 20 were >100-fold.

**TABLE 1:** Total numbers of differentially methylated peaks and relative genes.

Item	Up-methylated peak	Up-methylated gene	Down-methylated peak	Down-methylated gene
mRNA	3587	2790	850	671

**TABLE 2:** Top ten up-regulated methylated m^6^A-modified RNAs.

Chromosome	Start	End	Gene name	Log2(fold enrichment) *
chr21	45593654	45595448	LINC01694	12.10
chr16	29457604	29461526	SULT1A4	8.07
chr16	29457606	29458219	SLX1B	7.29
chr2	43233281	43233394	THADA	6.56
chr6	145629086	145629386	EPM2A	6.32
chr15	34528020	34528647	MIR1233-2	6.06
chr12	54146866	54147225	AC023794	6.02
chr4	80079856	80080180	ANTXR2	5.98
chr21	5026457	5028091	FP565260	5.98
chr17	81304454	81304988	LINC00482	5.86

*HL60/ADR vs. HL60.

**TABLE 3:** Top ten down-regulated methylated m^6^A-modified RNAs.

Chromosome	Start	End	Gene name	Log2(fold enrichment) *
chr17	46640120	46674590	NSF	-9.21
chr19	38251266	38251443	PPP1R14A	-6.27
chr1	118884304	118884484	TBX15	-5.32
chr5	70983858	70984097	NAIP	-5.32
chr2	100970857	100974825	NPAS2	-5.25
chr14	76263359	76263474	AC016526	-5.17
chr17	46514430	46514639	LRRC37A2	-5.17
chr5	70985504	70985923	NAIP	-5.11
chr7	144187372	144187762	ARHGEF35	-5
chr17	1936439	1937036	RTN4RL1	-4.70

*HL60/ADR vs. HL60.

KEGG (Kyoto Encyclopedia of Genes and Genomes) pathway analysis revealed that hyper-methylated m^6^A-modified genes in HL60/ADR were involved in the FoxO signaling pathway, while hypo-methylated m^6^A-modified genes were enriched in Notch signaling pathways, RNA transport, RNA degradation, and ribosome biogenesis in eukaryotes (Figures 2D,E).

Then, we analyzed fold enrichment of m^6^A peaks in different locations of mRNA between IP and input samples in both HL60 and HL60/ADR. The results showed that fold enrichment of m^6^A peaks in 5′UTR was higher than that in 3′UTR, and fold enrichment of m^6^A peaks in CDS was higher than that in both 5′UTR and 3′UTR in HL60 and HL60/ADR cells (Figure 2F). When comparing fold enrichment of m^6^A peaks in different locations of mRNA between HL60 and HL60/ADR, the results showed that fold enrichment of m^6^A peaks in 5′ UTR was higher in HL60/ADR than that in HL60. Similar results could be found in CDS and 3′ UTR of mRNA (Figure 2F). Global analysis of fold enrichment between HL60 and HL60/ADR indicated that fold enrichment of m^6^A peaks increased in HL60/ADR compared with that in HL60 (Figure 2G).

### Analysis of Differentially Expressed Genes by RNA-Seq

**FIGURE 3:**
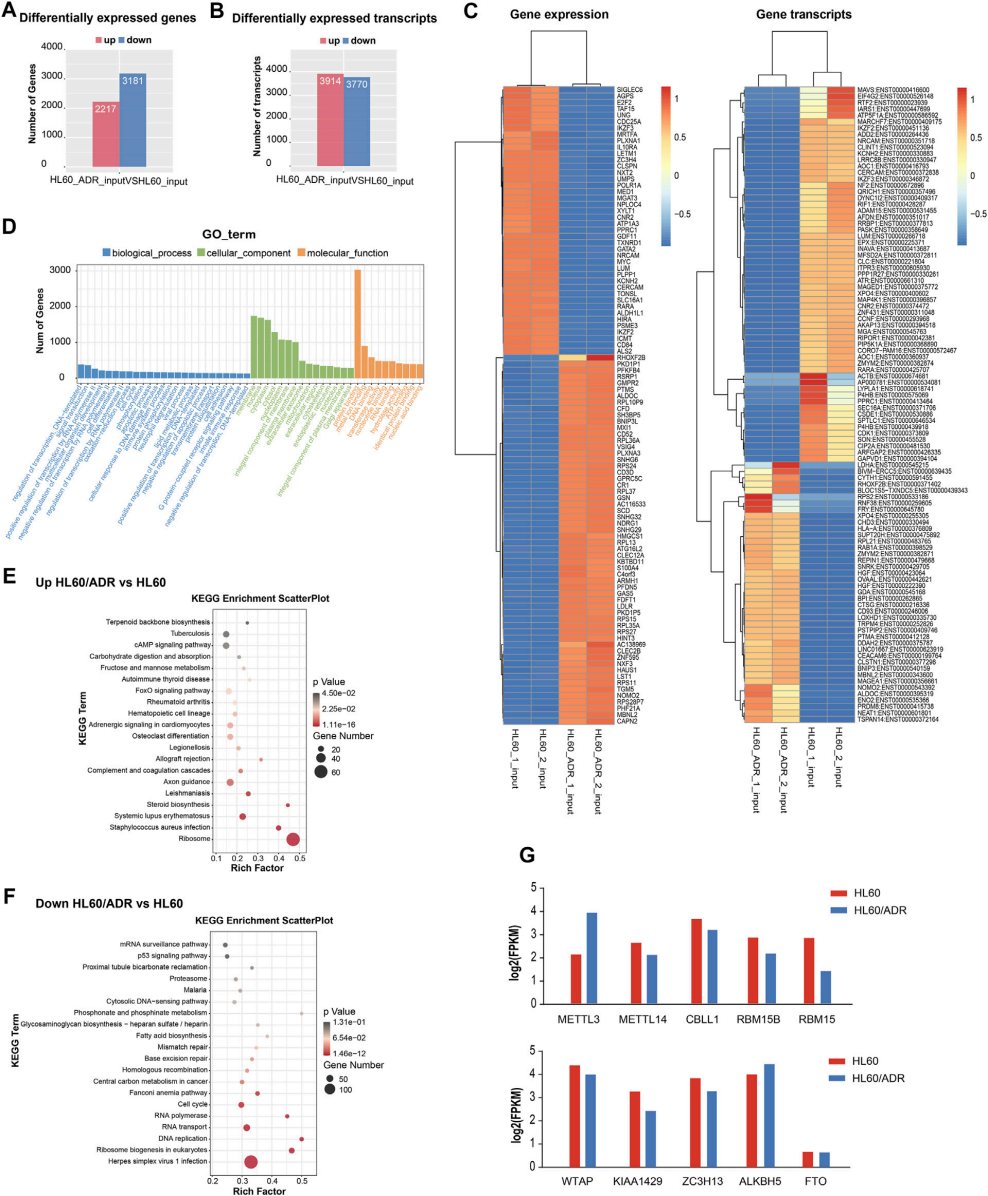
Analysis of differentially expressed genes by RNA-seq. **(A)** Numbers of differentially expressed genes. **(B)** Numbers of differentially expressed transcripts. **(C)** Heat map of differentially expressed genes and transcripts. **(D)** GO analysis included analysis of the biological process, cellular component, and molecular function. GO, Gene Ontology. **(E,F)** Pathway analysis of up-regulated genes **(E)** and down-regulated genes **(F)**. Rich factor, significant gene number/background gene number. KEGG, Kyoto Encyclopedia of Genes and Genomes. **(G)** Analysis of the expression level of ten m^6^A writers and erasers by RNA-seq in HL60 and HL60/ADR.

By analyzing RNA-seq results of the input library between HL60 and HL60/ADR, we detected a total of 5,398 differentially expressed genes, of which 2,217 were up-regulated and 3,181 were down-regulated. We also discovered 3,914 up-regulated transcripts and 3,770 down-regulated transcripts (Figures 3A,B). The heat map of differentially expressed genes is shown in Figure 3C.

Gene Ontology (GO) analysis revealed that the biological process was mainly enriched in the regulation of transcription and signaling transduction process. The cellular component was mainly enriched in the membrane, nucleus, cytoplasm. The molecular function was mainly enriched in protein binding function (Figure 3D). KEGG pathway analysis showed that up-regulated genes were enriched in the FoxO signaling pathway and the ribosome pathway (Figure 3E), and down-regulated genes were enriched in the p53 signaling pathway and ribosome biogenesis in eukaryotes (Figure 3F).

To analyze the expression level of ten m^6^A writers and erasers in RNA-seq data, we found an increase in METTL3 and ALKBH5 gene expression levels and a decrease in other m^6^A-related enzymes in HL60/ADR cells compared with HL60 cells (Figure 3G).

### Conjoint Analysis of MeRIP-Seq and RNA-Seq Data

**FIGURE 4:**
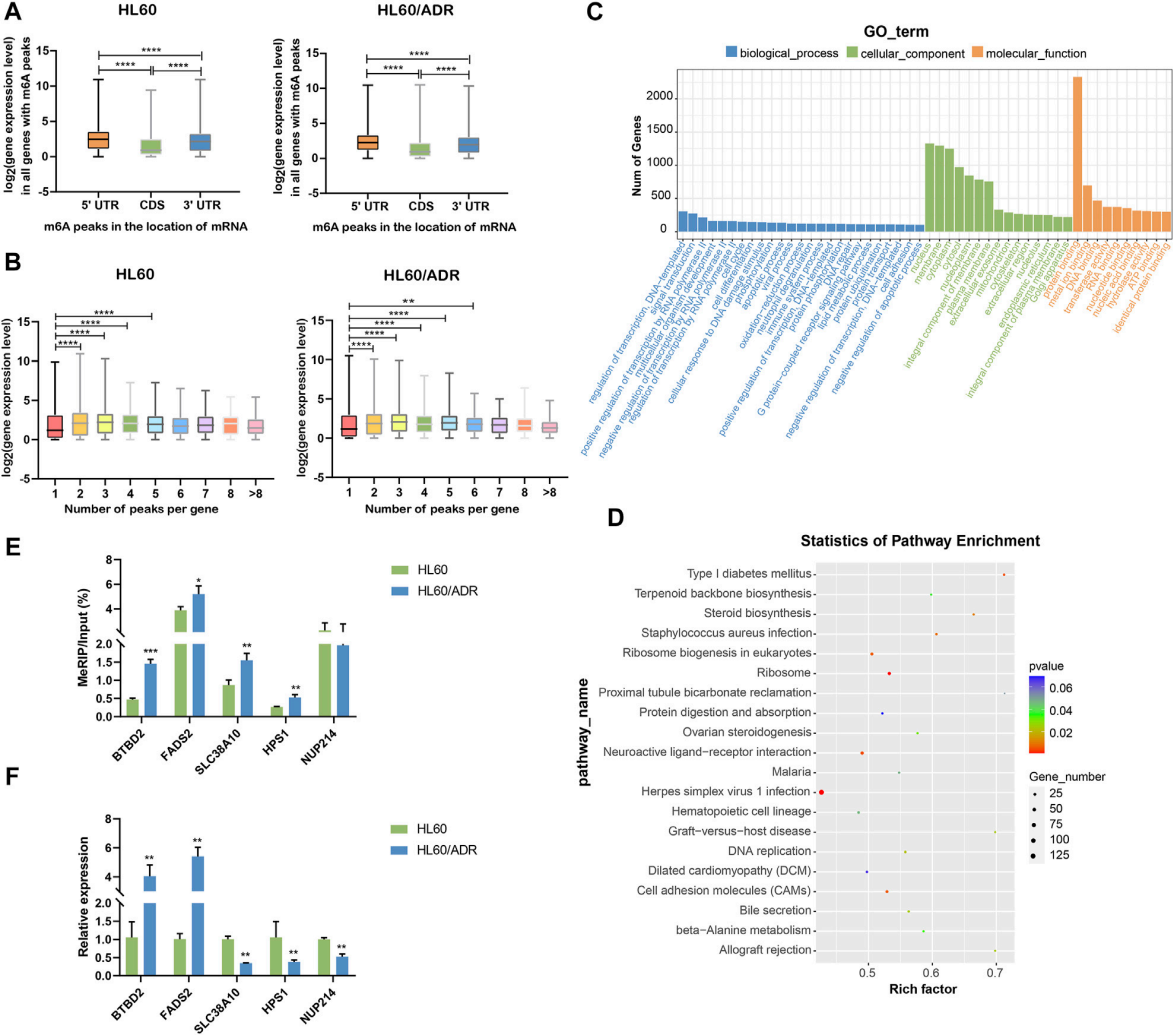
Conjoint analysis of MeRIP-seq and RNA-seq data. **(A)** Analysis of the expression level of all genes with m^6^A modification in different locations of mRNA. ****p<0.0001. **(B)** Analysis of the expression level of genes with a different number of m^6^A peaks. ***p<0.001, ****p<0.0001. **(C)** GO analysis of differentially expressed genes with m^6^A modification. GO, Gene Ontology. **(D)** KEGG pathway analysis of differentially expressed genes with m^6^A modification. Rich factor, significant gene number/background gene number. KEGG, Kyoto Encyclopedia of Genes and Genomes. **(E)** Validation of the m^6^A level by m^6^A IP-qPCR in five genes with differential m^6^A modification. Each group had three biological replicates. **(F)** Validation of the mRNA level by RT-qPCR in five genes with differential mRNA levels. Each group had three biological replicates.

By analyzing the global expression level of genes with m^6^A modification in different locations of mRNA, we found that the expression level of genes with m^6^A peaks in CDS was lower than that in 5′UTR and 3′UTR. The expression level of genes with m^6^A peaks in 5′UTR was higher than that in 3′UTR in both HL60 and HL60/ADR cells (Figure 4A). Then, we wanted to know whether the number of m^6^A peaks was associated with gene expression level. Results showed that genes with more than one m^6^A peak seemed to have higher expression levels compared with genes with one m^6^A peak (Figure 4B), similar to the results of Chen et al. (2020).

GO analysis revealed important roles of regulation of transcription and signaling transduction played in biological process, nucleus, membrane and cytoplasm part played in a cellular component, and protein binding function played in molecular function. KEGG analysis showed that differentially expressed genes were mainly enriched in ribosome and ribosome biogenesis in eukaryotes (Figures 4C,D).

To further verify our MeRIP-seq data, we selected five genes with differential m^6^A modification, including BTBD2, FADS2, SLC38A10, HPS1, and NUP214. Then, we performed m^6^A IP-qPCR with gene-specific primers. Results showed that changes in m^6^A levels were similar to MeRIP-seq data in four out of five genes (80%, Figure 4E, Supplementary Table S4), suggesting the reliability of our sequencing data. Analysis of mRNA levels of these genes showed the same tendency as RNA-seq data (Figure 4F, Supplementary Table S4).

### Elevated Global m^6^A Level in HL60/ADR Cells and Prognostic Value of m^6^A-Related Enzymes in AML Patients

**FIGURE 5:**
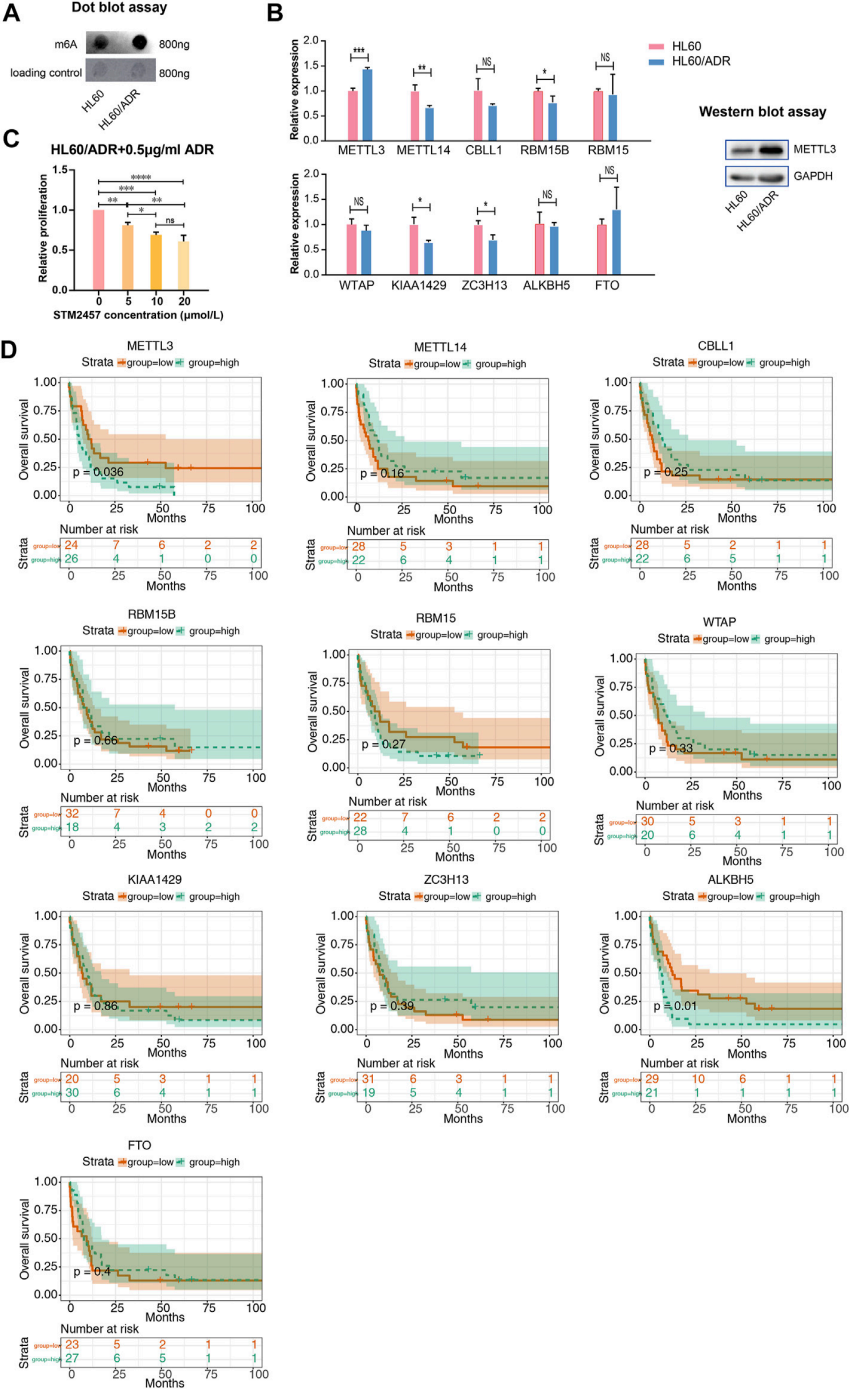
Analysis of the global m^6^A level and m^6^A-related enzymes. **(A)** Dot blot assay revealed a higher global m^6^A level in HL60/ADR than that in HL60. **(B)** Analysis of the expression level of ten m^6^A writers and erasers by RT-qPCR in HL60 and HL60/ADR. Western blot assay showed a higher protein level of METTL3 in HL60/ADR cells. *p<0.05, **p<0.01, ***p<0.001. NS, no significance. **(C)** Results of CCK-8 assay after treatment of STM2457 and ADR for three days. NS, no significance, *p<0.05, **p<0.01, ***p<0.001, ****p<0.0001. Each group had three biological replicates. **(D)** Overall survival analysis of ten m^6^A writers and erasers in AML patients.

We performed a global analysis of m^6^A levels in HL60 and HL60/ADR cells. The result of dot blot experiment showed that the global m^6^A level in HL60/ADR was higher than that in HL60 (Figure 5A). To study the reason why the global m^6^A level elevated in ADR-resistant AML cells, we explored ten m^6^A-related enzymes, including METTL3, METTL14, CBLL1, RBM15B, RBM15, WTAP, KIAA1429, ZC3H13, ALKBH5, and FTO (Zhang et al., 2020). RT-qPCR results revealed that METTL3 expression level increased in HL60/ADR cells, while the expression level of METTL14, RBM15B, KIAA1429, and ZC3H13 decreased in HL60/ADR (Figure 5B). Western blot experiment further confirmed that the protein expression level of METTL3 was higher in HL60/ADR than in HL60 (Figure 5B). Then, we treated HL60/ADR cells with different concentrations of STM2457 (0, 5, 10, and 20 μmol/L) combined with ADR (0.5 μg/ml) for three days, the half-maximal inhibitory concentration (IC50) of STM2457 was 24.15 μmol/L, and the IC50 of ADR was 2.87 μg/ml. The IC50 of STM2457 in combined therapy was 22.29 μmol/L (Supplementary Figure S1). Results showed that the proliferation of HL60/ADR was inhibited in a dose-dependent manner (Figure 5C).

We then analyzed the prognostic value of m6A-related enzymes in AML patients in TCGA database. Clinical data and RNA-seq information of 163 AML patients were downloaded from TCGA database. Clinical characteristics of all AML patients are shown in Supplementary Table S5. Patients were divided into two groups based on the median gene expression level. We found that higher levels of METTL3 and ALKBH5 related to adverse overall survival rates in cytogenetic poor-risk patients (Figure 5D). Clinical characteristics of cytogenetic poor-risk AML patients are shown in Supplementary Table S6. In general, METTL3 was elevated in ADR-resistant AML cells and played a prognostic value in cytogenetic poor-risk AML patients.

## Discussion

M^6^A methylation is an important modification of mRNA critical for mRNA stability, splicing, transport, and translation (Tuck, 1992; Meyer et al., 2015; Xiao et al., 2016; Huang et al., 2018). It plays important roles in various pathological processes, including initiating, development, progression, and drug resistance of cancers (Deng et al., 2018). Here, we analyzed transcriptome-wide m^6^A distribution in HL60 and HL60/ADR cells.

In our study, we found that the m^6^A modification pattern in HL60/ADR was slightly different from that in HL60. We discovered that the numbers of unique m^6^A-modified genes (Figure 1B) and m^6^A-modified peaks (by selection criteria of *p*<0.05) in HL60/ADR were lower than in HL60. We observed that percentage of genes with one m^6^A peak was lower in HL60/ADR than in HL60, whereas the percentage of genes with two to six m^6^A peaks was slightly higher in HL60/ADR than that in HL60 (Figure 1E). In addition, we found that over 40% of genes had one m^6^A peak, and the percentage of genes with two or more m^6^A peaks was lower than that with only one m^6^A peak (Figure 1E). Nonetheless, the expression level of genes with more than one m^6^A peak seemed higher than that of genes with one m^6^A peak (Figure 4B). These might reveal that the distribution of m^6^A modification in genes was different, and the relationship between m^6^A modification and gene expression was complex. Furthermore, the function of m^6^A readers (Wang et al., 2015; Roundtree et al., 2017; Shi et al., 2017; Huang et al., 2018; Mao et al., 2019), which identify m^6^A modification and are crucial to m^6^A recognition and determinate the fate of mRNA, increase the complexity of m^6^A regulation.

By analyzing differentially methylated and expressed genes, we found pathways were enriched in the FoxO signaling pathway, p53 signaling pathway, and Notch signaling pathway (Figures 2D,E, Figures 3E,F). Research has shown that the aberrant expression of FOXOs was responsible for drug resistance. Continuous activation of FOXO3 led to drug resistance in CML cells after tyrosine kinase inhibitor or imatinib therapy (Hui et al., 2008a; Hui et al., 2008b; Naka et al., 2010). In FLT3-ITD^+^ AML cells resistant to FLT3 inhibition, Long demonstrated that histone deacetylase 8 (HDAC8) up-regulated through FOXO1- and FOXO3-mediated transactivation, leading to inactivation of the p53 pathway. Inhibition of HDAC8 reactivated p53 and significantly enhanced the TKI-mediated anti-FLT3-ITD^+^ AML cells (Long et al., 2020). Another study showed that activation of p53 might overcome the resistance of FLT3-ITD AML cells to FLT3 inhibitors (Park et al., 2017). Wang also found that an inhibitor of histone deacetylase 6 (HDAC6) could reverse ADR resistance in human breast cancer cells by activating the p53 pathway (Wang et al., 2018). Feng showed that annexin A2 induced cisplatin resistance of NSCLCs *via* suppressing the expression of p53 (Feng et al., 2017). In addition, the Notch signaling pathway was reported to be associated with drug resistance in breast cancer, lung adenocarcinoma, myeloma, and other malignant lymphoid cell lines (Nefedova et al., 2004; Yan et al., 2018; Bousquet Mur et al., 2020). All these findings suggested that the FoxO signaling pathway, p53 signaling pathway, and Notch signaling pathway might play crucial roles in drug resistance of tumors. Further studies should be conducted to study whether there is an association between these pathways and ADR resistance in AML.

A comparison of the global m^6^A level between HL60 and HL60/ADR showed that it was elevated in HL60/ADR. Fold enrichment of m^6^A peaks was higher in HL60/ADR than that in HL60 (Figure 2G), which indicated that the m^6^A level was relatively higher in HL60/ADR by MeRIP-seq. The result was further confirmed by dot blot assay (Figure 5A). We also found that fold enrichment of m^6^A peaks in 5′ UTR, CDS, or 3′ UTR of mRNA in HL60/ADR was higher than that in HL60 (Figure 2F), which might cause an overall elevated level of m^6^A peaks in HL60/ADR. By analyzing the reason for the elevated global m^6^A level in HL60/ADR, we detected the expression level of ten m^6^A-related enzymes. RNA-seq results showed an increase of METTL3 expression level in HL60/ADR (Figure 3G), which was further confirmed by RT-qPCR (Figure 5B). Western blot assay also showed an increase of METTL3 protein level in HL60/ADR (Figure 5B). After a combined treatment of STM2457 and ADR for three days, the proliferation of HL60/ADR was inhibited (Figure 5C). All these might suggest an association between ADR resistance and METTL3. METTL3 has been reported to be involved in the drug resistance of some other malignancies (Jin et al., 2019; Liu et al., 2020; Lai et al., 2021; Pan et al., 2021). The mechanism of METTL3 in ADR resistance of AML is still needed to be further investigated. Overall survival analysis of cytogenetic poor-risk AML patients revealed that patients with a higher level of METTL3 had an inferior prognosis (Figure 5D), which suggested a prognostic value of METTL3 in poor-risk AML patients. As we know, relapsed/refractory AML patients also have a poor prognosis. Whether METTL3 plays a prognostic value in relapsed/refractory AML patients is still needed to be investigated. In summary, ADR resistance in AML might associate with abnormal m^6^A modification.

## Conclusion

This study presented the global m^6^A pattern of ADR-resistant AML cells. The global m^6^A level was elevated in HL60/ADR. The expression level of METTL3 was higher in ADR-resistant AML cells. A combined treatment of METTL3 inhibitor and ADR inhibited cell proliferation in ADR-resistant AML cells. All these might suggest that the abnormality of m^6^A modification played an important role in Adriamycin-resistant AML.

## Data Availability

The public gene expression and patients’ clinical information dataset can be downloaded from TCGA database (https://www.cancer.gov/about-nci/organization/cc-g/research/structural-genomics/tcga). The data of MeRIP-seq are available on the GEO website (https://www.ncbi.nlm.nih.gov/geo/query/acc.cgi?acc=GSE192618). Other data are included in the article/Supplementary Material.
