# Supplementary material for: Transcriptome-Wide Analysis of RNA N6-Methyladenosine Modification in Adriamycin-Resistant Acute Myeloid Leukemia Cells
Source: Front Genet. 2022 Apr 28;13:833694. doi: 10.3389/fgene.2022.833694 (PMC9100953; doi:10.3389/fgene.2022.833694)
Supplement: Supplementary file 1 [file Table1.docx]

**Supplementary Table S1. Specific primers for each evaluated gene.**

| **Gene name** | **Forward primers sequences** | **Reverse primers sequences** |
| --- | --- | --- |
| METTL3 | ATCCCCAAGGCTTCAACCAG | GCGAGTGCCAGGAGATAGTC |
| METTL14 | AGAGAACAAAGGAACACTGCCT | AATGAAGTCCCCGTCTGTGC |
| CBLL1 | AAGATGTGTCCAGGCTGTAGT | TGTGCTGCTTTGGCGGAATA |
| RBM15B | TACACGGAGGCTACCAGTACA | GTCGTACAGCCCGTAGTAGTC |
| RBM15 | ACGACCCGCAACAATGAAG | GGAAGTCGAGTCCTCACCAC |
| WTAP | GCTTCTGCCTGGAGAGGATT | TGCAGACTCCTGCTGTTGTT |
| KIAA1429 | CTTGGCAAGTGGCTTGAACC | ACGTAAGGCAGTGGTAAGGC |
| ZC3H13 | CGCTGTGAAGCAAAACTGGA | ACTCGTCATCTCCTGCTTCC |
| ALKBH5 | CGGCGAAGGCTACACTTACG | CCACCAGCTTTTGGATCACCA |
| FTO | ACTTGGCTCCCTTATCTGACC | TGTGCAGTGTGAGAAAGGCTT |
| GAPDH | GGAGCGAGATCCCTCCAAAAT | GGCTGTTGTCATACTTCTCATGG |

**Supplementary Table S2. Gene-specific primers for m^6^A IP-qPCR.**

| **Gene name** | **Forward primers sequences** | **Reverse primers sequences** |
| --- | --- | --- |
| BTBD2 | CCGTCCTGATGCTGAGAA | CCGAGGTCATCTTCTACAC |
| FADS2 | AGCGCGAGGTGTCGGTG | CGCGGAAGGCATCCGTTG |
| SLC38A10 | CAGTTGGCTTCCTTCAGA | AATTCCCTGGGCAAAGTC |
| HPS1 | CTGGAAGAGGAGCTGTTG | TGAGGATAGGGCAGAGTG |
| NUP214 | CACCAGCACTTCCTCAAC | GCCAGCACTTAGAGATGTC |

**Supplementary Table S3. Samples and quality control information.**

| **Sample_ID** | **Raw_Reads** | **Raw_Bases** | **Valid_Reads** | **Valid_Bases** | **Valid%** | **Q20%** | **Q30%** | **GC%** |
| --- | --- | --- | --- | --- | --- | --- | --- | --- |
| HL60_1_IP | 47063456 | 7.06G | 45613156 | 6.26G | 88.64 | 97.84 | 93.46 | 49.61 |
| HL60_ADR_1_IP | 51366896 | 7.71G | 50174360 | 6.91G | 89.64 | 97.82 | 93.45 | 50.61 |
| HL60_1_input | 51750814 | 7.76G | 50796044 | 6.97G | 89.79 | 97.54 | 92.78 | 49.27 |
| HL60_ADR_1_input | 41697474 | 6.25G | 41255722 | 5.68G | 90.88 | 97.51 | 92.74 | 49.19 |
| HL60_2_IP | 53745092 | 8.06G | 52094144 | 7.17G | 88.91 | 97.26 | 92.26 | 49.50 |
| HL60_ADR_2_IP | 55630126 | 8.34G | 54314882 | 7.50G | 89.86 | 97.10 | 91.98 | 50.47 |
| HL60_2_input | 51139700 | 7.67G | 50254544 | 6.91G | 90.15 | 97.54 | 92.89 | 49.19 |
| HL60_ADR_2_input | 54663830 | 8.20G | 54116914 | 7.48G | 91.18 | 97.54 | 92.86 | 49.08 |

**Supplementary Table S4. List of five genes with differential m^6^A modification and mRNA levels.**

| Gene name | Chromosome | m^6^A level change | | | mRNA level change | |
| --- | --- | --- | --- | --- | --- | --- |
|  |  | Peak region | Log2 (Fold change) | p-value | Fold change | p-value |
| BTBD2 | chr19 | 3' UTR | 3.74 | 0.001 | 4.221 | 7.8E-192 |
| FADS2 | chr11 | 3' UTR | 3.75 | 0.078 | 7.272 | 0 |
| SLC38A10 | chr17 | 3' UTR | 3.81 | 0.033 | 0.286 | 2.6E-118 |
| HPS1 | chr10 | 5' UTR | 3.17 | 0.021 | 0.452 | 8.97E-36 |
| NUP214 | chr9 | Exon | -3.51 | 0.022 | 0.339 | 3.1E-91 |

**Supplementary Table S5. Clinical characteristics of 163 non-M3 AML patients in TCGA database.**

| Characteristics | No. | % |
| --- | --- | --- |
| Age, y, median (range) | 58 (18—88) | — |
| Gender, no |  |  |
| Female | 76 | 46.6 |
| Male | 87 | 53.4 |
| Median WBC, ×10^9^/L (range) | 19.9 (0.6—297.4) | — |
| Median BM blast, % (range) | 72 (30—100) | — |
| FAB classification |  |  |
| M0 | 16 | 9.8 |
| M1 | 44 | 27.0 |
| M2 | 40 | 24.5 |
| M4 | 35 | 21.5 |
| M5 | 21 | 12.9 |
| M6 | 2 | 1.2 |
| M7 | 3 | 1.8 |
| Unknown | 2 | 1.2 |
| Cytogenetic risk classification |  |  |
| Favorable | 18 | 11.0 |
| Intermediate | 92 | 56.4 |
| Poor | 50 | 30.7 |
| Unknown | 3 | 1.8 |
| Transplant |  |  |
| No | 85 | 52.1 |
| Yes | 78 | 47.9 |

**Supplementary Table S6. Clinical characteristics of 50 cytogenetic poor-risk AML patients in TCGA database.**

| Characteristics  Patients number | High METTL3 (≤ median)  N = 24 | Low METTL3 (>median)  N = 26 | *p-*value |
| --- | --- | --- | --- |
| Age, y, median (range) | 53.5 (18.0—81.0) | 63 (39—81) | 0.066 |
| Gender, no. (%) |  |  | 0.069 |
| Female | 6 (25.0) | 13 (50.0) |  |
| Male | 18 (75.0) | 13 (50.0) |  |
| Median WBC, ×10^9^/L (range) | 10.3 (0.8—90.4) | 16.5 (0.7—171.9) | 0.256 |
| Median BM blast, % (range) | 71.5 (30.0—97.0) | 74.5 (32.0—95.0) | 0.801 |
| FAB classification, no. (%) |  |  | 0.219 |
| M0 | 2 (8.3) | 7 (26.9) |  |
| M1 | 5 (20.8) | 6 (23.1) |  |
| M2 | 4 (16.7) | 7 (26.9) |  |
| M4 | 5 (20.8) | 2 (7.7) |  |
| M5 | 6 (25.0) | 2 (7.7) |  |
| M6 | 1 (4.2) | 0 (0) |  |
| M7 | 1 (4.2) | 1 (3.8) |  |
| Unknown | 0 (0) | 1 (3.8) |  |
| Transplant, no. (%) |  |  | 0.258 |
| No | 10 (41.7) | 15 (57.7) |  |
| Yes | 14 (58.3) | 11 (42.3) |  |


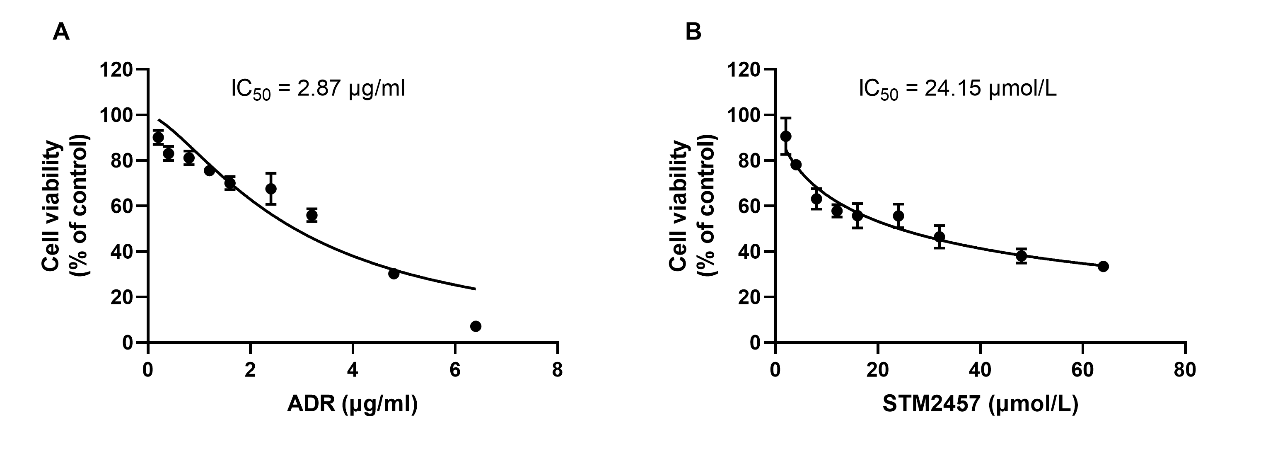


**Supplementary Figure S1.** Dose response curves for HL60/ADR cells with the treatment of Adriamycin (A) and STM2457 (B) for three days. ADR, Adriamycin. Each group had three biological replicates.
